# Methodology for tissue sample collection within a translational sub-study of the CHHiP trial (CRUK/06/016), a large randomised phase III trial in localised prostate cancer

**DOI:** 10.1016/j.ctro.2018.02.002

**Published:** 2018-02-16

**Authors:** Anna Wilkins, Christine Stuttle, Shama Hassan, Claire Blanchard, Clare Cruickshank, Clare Griffin, Jake Probert, Catherine M Corbishley, Chris Parker, David Dearnaley, Emma Hall

**Affiliations:** a The Institute of Cancer Research, 123 Old Brompton Road, London SW7 3RP, United Kingdom; b Royal Marsden Hospital, Downs Road, Sutton SM2 5PT, United Kingdom

**Keywords:** BATS, Blood and Tissue Samples database, BIDD, Biomarker and Imaging Discovery and Development Committee, CHHiP, Conventional or Hypofractionated High dose intensity modulated radiotherapy in Prostate cancer, CRN, Clinical Research Network, CTU, Clinical Trials Unit, H&E, Haematoxylin and Eosin, ICR-CTSU, Institute of Cancer Research Clinical Trials and Statistics Unit, ISUP, International Society of Urological Pathology, MTA, Material Transfer Agreement, NCCN, National Comprehensive Cancer Network, NCRI, National Cancer Research Institute, NHS, National Health Service, TMA, Tissue microarray, TMG, Trial Management Group, TSC, Trial Steering Committee, TURP, Trans-urethral resection of prostate, Sample collection methodology, Prostate cancer biopsies, Translational study

## Abstract

**Background:**

This article presents the methodology for tissue sample collection in Trans-CHHiP, the main translational study within the CHHiP (Conventional or Hypofractionated High dose intensity modulated radiotherapy in Prostate cancer, ISRCTN 97182923) trial. The CHHiP trial randomised 3216 men with localised prostate cancer to 3 different radiotherapy fractionation schedules. Trans-CHHiP aims to identify biomarkers of fraction sensitivity.

**Methods:**

We outline the process of tissue collection, including central review by a study-specific specialist uropathologist and comparison of the centrally-assigned Gleason grade group with that assigned by the recruiting-centre pathologist.

**Results:**

2047 patients provided tissue from 107 pathology departments between August 2012 and April 2014. A highly motivated Clinical Trials Unit chasing samples and a central Trans-CHHiP group that regularly reviewed progress were important for successful sample collection. Agreement in Gleason grade group assigned by the recruiting centre pathologist and the central study-specific uropathologist occurred in 886 out of 1854 (47.8%) cases. Key lessons learned were the need for prospective consent for tissue collection when recruiting patients to the main trial, and the importance of Material Transfer Agreement (MTA) integration into the initial trial site agreement.

**Conclusions:**

This methodology enabled collection of 2047 patient samples from a large randomised radiotherapy trial. Central pathological review is important to minimise subjectivity in Gleason grade grouping and the impact of grade shift.

## Highlights

• This article presents the methodology for tissue sample collection in the CHHiP trial.
• 2047 patients provided tissue from 107 pathology departments between August 2012 and April 2014.
• Central pathological review was important to minimise subjectivity in Gleason grade grouping and the impact of grade shift.
• A key lesson learned was the need for prospective consent for tissue collection at trial recruitment.
• Material Transfer Agreement (MTA) integration into the initial trial site agreement is important.

## Background

The progression towards personalised medicine requires development and validation of robust predictive biomarkers. Phase III clinical trials provide an excellent opportunity to conduct translational biomarker studies as large numbers of patients with similar disease characteristics are randomised to different interventions and outcome data are collected prospectively through standard proforma. Efficient collection of patient samples is an obvious pre-requisite for biomarker studies and presents logistical challenges which are not well-represented in the published literature. Identifying strengths and weaknesses of methodologies for sample collection is increasingly important as technological innovation and improved understanding of tumour biology offer increased potential for introduction of biomarkers to routine clinical care.

The CHHiP trial is a phase III non-inferiority trial that recruited 3216 men with localised prostate cancer from 71 centres to radiotherapy treatment between 2002 and 2011 [1]. Most men recruited to CHHiP had intermediate risk localised prostate cancer, a risk category where biochemical recurrence varies considerably from 10% to 40% [2], and our understanding of how to stratify patients is limited. Additionally, CHHiP is the largest trial of different radiotherapy fractionation schedules for prostate cancer to date; therefore it provides a unique opportunity for translational work to improve our understanding of the biological basis of fraction sensitivity.

Trans-CHHiP (CRUK A12518: An evaluation of biomarkers in hypofractionated and dose escalated prostate cancer radiotherapy) was established as the main translational study within the CHHiP trial. It aims to identify biomarkers of fraction sensitivity and improve risk stratification for patients with intermediate risk localised prostate cancer. Patient tissue samples were collected between 2012 and 2016, this article presents the methodology used for sample collection in Trans-CHHiP, together with lessons learned that could improve efficiency of sample collection in the future.

## Methods

### Study organisation

#### Trans CHHiP central group

Sample collection from participating centres was coordinated by a central Trans-CHHiP group based at the Institute of Cancer Research (ICR). This group met at least 3 monthly throughout the sample collection process to review progress, resolve problems and plan new aspects of sample collection. The group included members of the ICR Clinical Trials and Statistics Unit (ICR-CTSU) CHHiP team (scientific lead, trial managers, trial administrator and clinical research fellow), the trial Chief Investigator, a dedicated biomedical scientist and a study-specific diagnostic uropathologist.

#### Recruiting centres

67 recruiting CHHiP centres (excluding 4 trial centres outside the UK) from 58 National Health Service (NHS) Trusts were eligible for Trans-CHHiP. The ICR-CTSU communicated directly with recruiting centre pathology departments for sample collection (see below). All CHHiP centres were updated about Trans-CHHiP progress via annual teleconferences.

#### CHHiP governance groups

The CHHiP trial is overseen by a Trial Management Group (TMG) which meets 6 monthly, during which Trans-CHHiP updates are provided. In addition external data access requests for use of biological and/or clinical data are reviewed by the TMG and Trial Steering Committee (TSC) as they arise. The TSC is an independent oversight group comprising clinical and statistical members.

#### Study specific databases e.g BATS, CHHiP Progeny

Two study-specific databases were created for Trans-CHHiP. Firstly the Blood and Tissue Samples database (BATS), which records the patient’s histology number, unique trial number, recruiting CHHiP centre and pathology department where the tissue was held. It also contains details of Trans-CHHiP consent, whether samples have been requested, received and whether invoices for samples have been paid.

Secondly a customised version of Progeny software including the Sample Management module was created for Trans-CHHiP. This includes 11 pathology variables, and the Gleason score. It details the precise physical location of each sample within the CHHiP inventory. Progeny enables 2D barcoding for all slides, cassettes and eppendorf tubes, and creation of 91 disc digital tissue microarray (TMA) plates.

### Patient consent, translational study contract and MTA

CHHiP was undertaken in 3 seamless stages with different consent for tissue donation between part I and parts II/III of the trial. For parts II/III consent for donation of tissue was included as an optional tick box clause in the main trial consent form. However, for Part I of the trial, this clause was not present and patients were re-consented (if possible) at a later date.

A Material Transfer Agreement (MTA) outlining terms and conditions of transfer of samples between academic organisations was required between the ICR and any NHS Trust donating tissue to Trans-CHHiP.

### Funding and financial reimbursement

Sample collection was funded by a Cancer Research UK Biomarkers and Imaging Discovery and Development Committee (BIDD) grant (A12518) obtained in 2011. Cancer Research UK reimbursement to pathology departments was £15 per patient; having provided tissue, these departments submitted invoices directly to the ICR-CTSU. Invoices submitted at a higher cost per sample were reviewed by the Chief Investigator and funded where feasible. As CHHiP was within the National Institute for Health Research Clinical Research Network (NCRN) portfolio, pathology departments were encouraged to approach the CRN to help with additional resources for sample collection. Further funding for the central receiving laboratory at the ICR was provided by Prostate Cancer UK and the Movember Foundation.

### Tissue collection process prior to arrival of tissue at central receiving laboratory

**Fig. 1:**
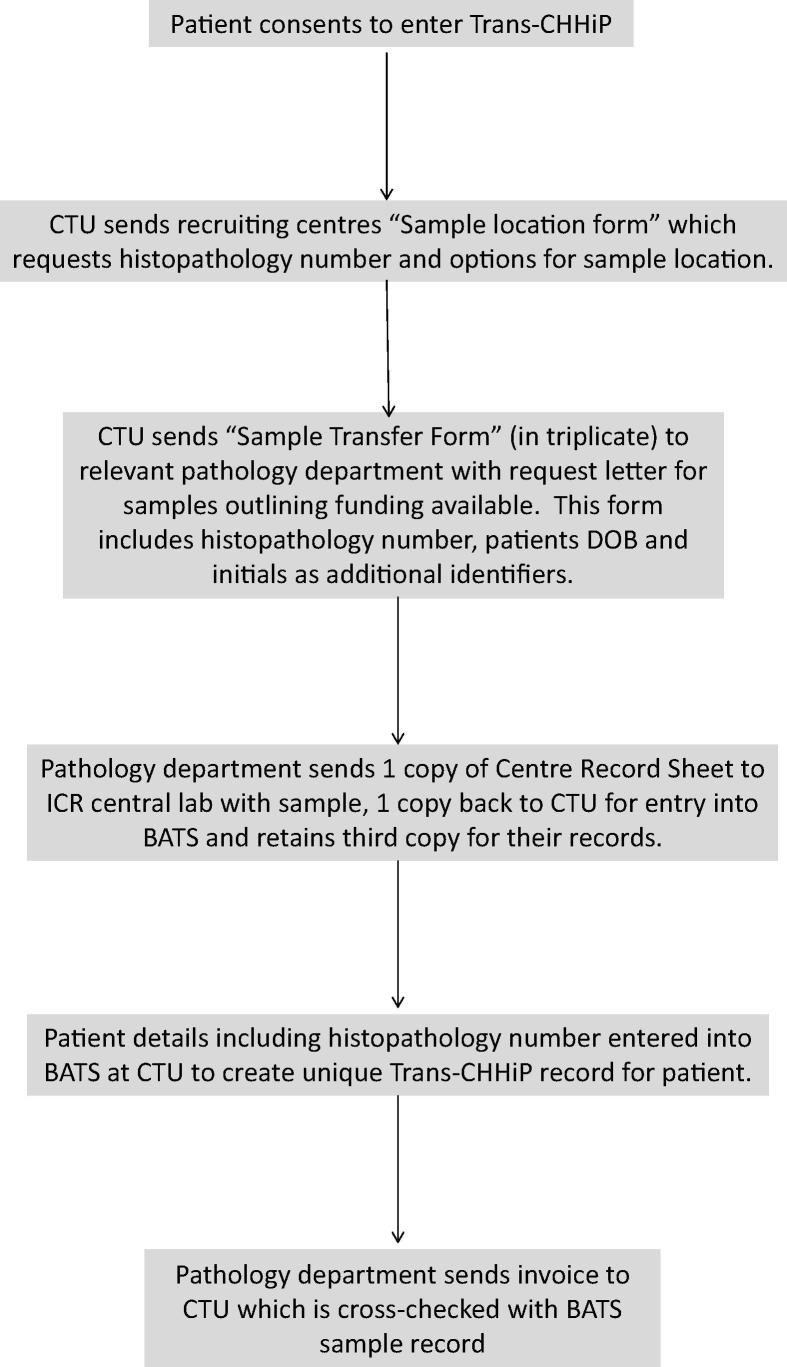
Sample collection process prior to arrival of sample at central receiving laboratory DOB: Date of birth, BATS: Blood and Tissue Samples database, CTU: Clinical Trial Unit.

The steps for sample collection prior to arrival at the central receiving laboratory are summarised in Fig. 1. As sample location was unknown to the CHHiP team, a patient level “Sample location form” was created to enable recruiting centres to indicate where the sample was stored. A “Sample Transfer Form” was also created to ensure consistent record of sample transfer between the donating histopathology department, the central receiving Trans-CHHiP laboratory and the ICR-CTSU. (See appendix for both forms.)

A key requirement prior to the ICR-CTSU sending sample request letters to centres was that the MTA was in place between the ICR and the relevant NHS Trust. Several NHS Trusts wanted to renegotiate the terms of the original site agreement; set up of the MTA was, in some cases, a costly and lengthy process that significantly delayed sample collection. Some NHS Trusts comprised more than one recruiting CHHiP centre and a total of 58 NHS Trusts included the 67 recruiting centres for Trans-CHHiP. All 58 NHS Trusts did eventually complete the MTA, however the time taken for completion ranged from 6 to 777 days. Issues that arose during MTA negotiation included return of samples, change of NHS Trust names and splitting and merging of NHS Trusts since the original agreement. Additionally, some hospital pathology departments would not release blocks before seeing the relevant signed consent form, which required the ICR-CTSU to obtain and transfer the relevant form. A number of pathology departments had archived samples off site which further complicated obtaining tissue.

ICR-CTSU had a systematic programme for chasing up requested samples with an initial letter and subsequent phone calls if centres did not respond within one month of the letter. A substantial proportion of centres needed reminders. On average 3 reminders were required, but for some centres up to 6 repeated requests were sent. Subsequent to receipt of samples, the ICR-CTSU proactively chased pathology departments for invoices. Once an invoice arrived, it was cross-checked with the BATS record of samples previously received prior to payment.

### Tissue collection process after arrival of tissue at central recruiting laboratory

**Fig. 2:**
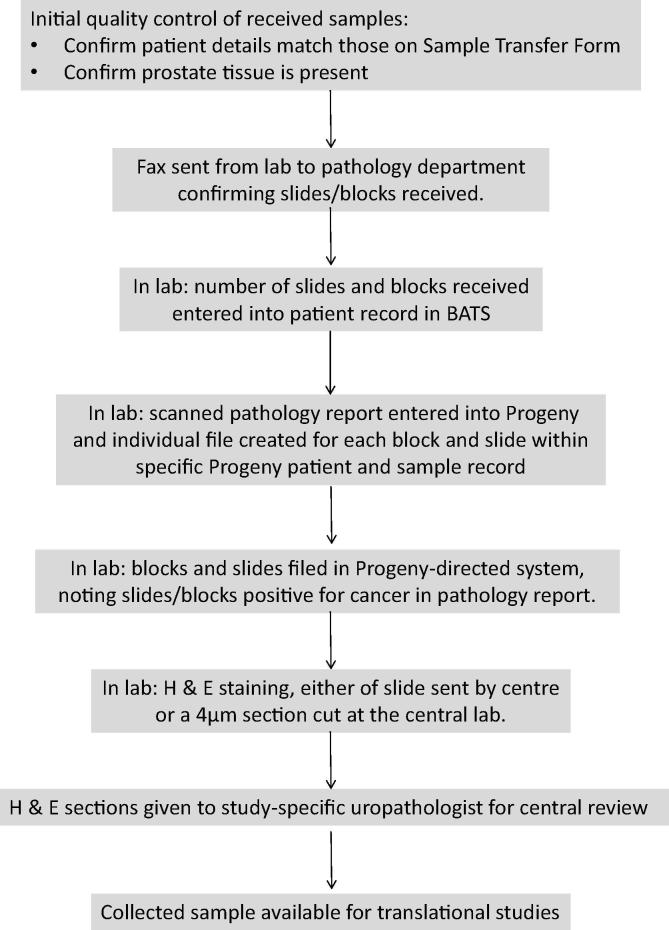
Sample collection process once sample has arrived at central receiving laboratory H & E: Haematoxylin and Eosin, BATS: Blood and Tissue Samples database.

The key steps in the tissue collection process once the sample had arrived at the ICR central laboratory are summarised in Fig. 2. All blocks and slides from the diagnostic sample were requested from each consenting patient. In some cases only blocks or slides were received, or just representative slide(s) and/or block(s). Both core biopsy specimens and trans-urethral resection of prostate (TURP) specimens were requested and reviewed. All samples were stored in a single laboratory at room temperature.

All cases were centrally reviewed by a specialist consultant uropathologist with an interest in prostate pathology. Cases were assessed using the ISUP grading recommendations first proposed in 2005, amendments to grading proposed in 2014 at the ISUP consensus conference were also incorporated [3], [4]. Grade shift in prostate cancer reporting in the last 10 years is well-recognised following the adoption of these standards by the majority of uropathologists worldwide. Tumour typing followed the recommendations of the WHO, subtypes such as invasive ductal adenocarcinoma were noted. The review process allowed the cases to be assigned grade groups according to the recent recommendations of ISUP and WHO [3], [4], [5], [6], [7].

**Fig. 3:**
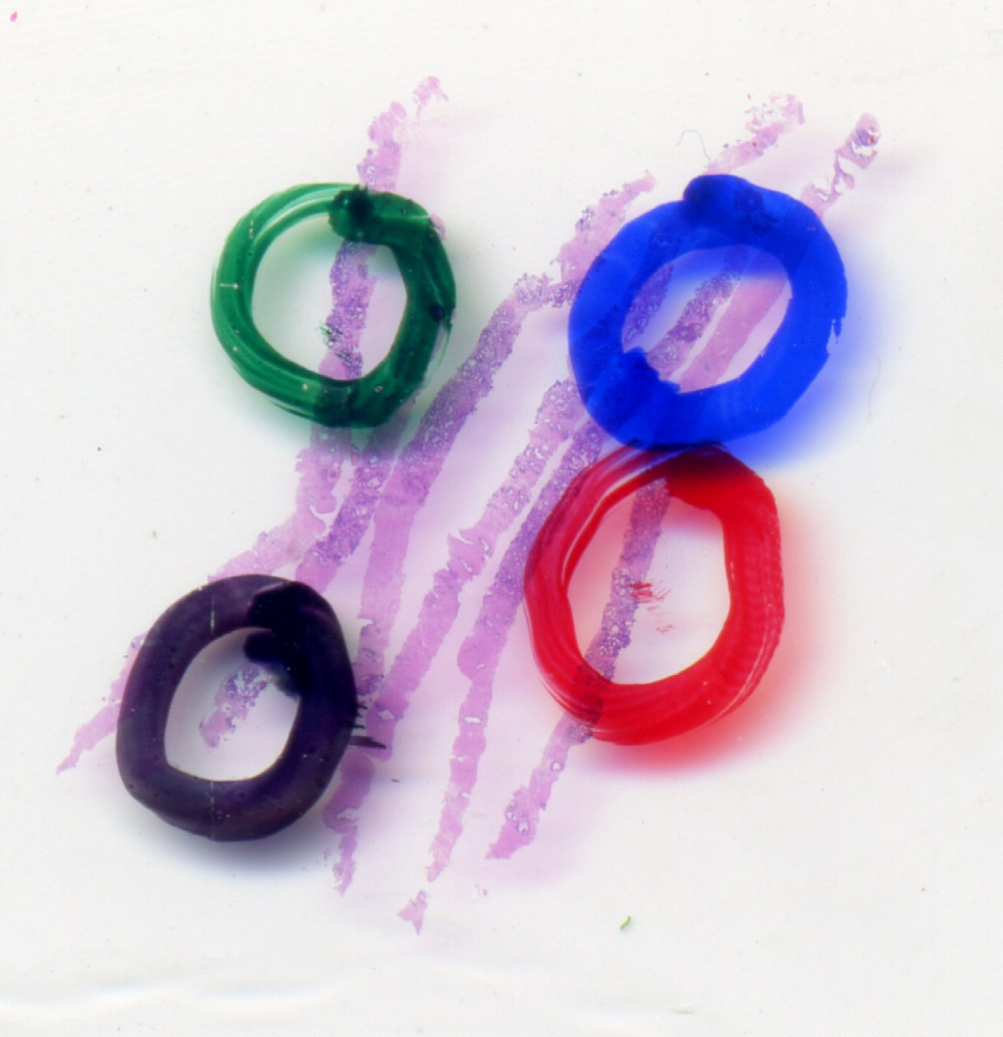
Core biopsies stained with H&E, maximum tumour cellularity areas marked by uropathologist. H&E: Haematoxylin and Eosin.

All available tumour slides were reviewed with the specialist uropathologist blinded to the origin of the samples and the original pathology report. Where possible the original Haematoxylin and Eosin (H & E) stained slides were examined but some blocks needed to be recut because slides were not submitted or were damaged. Each core biopsy was separately scored and an overall Gleason score given. Additional data such as maximum tumour length was also recorded. Up to four areas of representative tumour were marked on the slides using a colour code to indicate hierarchy of the most representative areas for further study (see Fig. 3). For TURP specimens the number and percentage of chips involved by tumour was assessed, an overall Gleason grade assigned and representative tumour areas marked as for core biopsies (see appendix for assessment forms).

Tissue microarray (TMA) using the checkerboard technique was initially planned as a high-throughput method for immunohistochemistry staining in Trans-CHHiP. TMA offers the advantage of tissue preservation, provided adequate tumour cellularity is present in the TMA [8]. To evaluate biopsy TMA (bTMA) use in Trans-CHHiP, a single bTMA was prepared by cutting 4 mm portions from the tumour blocks matching the red circles demarcated by the study-specific uropathologist. These portions were temporarily stored in eppendorf tubes at room temperature, before creation of the pilot bTMA using the checkerboard technique [8].

## Results and discussion

### Samples collected

**Fig. 4:**
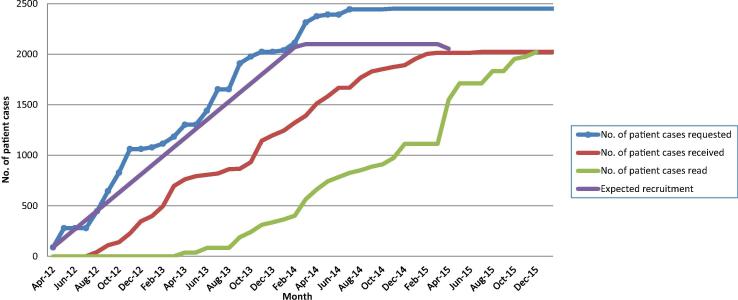
Chronological view of patient cases requested, received and read by study specific uropathologist.

2749/3179 UK CHHiP trial patients consented to tissue collection and 2451 requests for samples were issued to 123 hospitals. Between August 2012 and April 2014 samples were received for 2047 (83.5%) patients from 107 pathology departments, although for 40 patients only slides were provided (see table 1). Table 1 displays the number of blocks received per patient. The number of prostatic cores present in a single block varied from a single core to more than 10 cores per block. All samples were centrally reviewed by a specialist uropathologist between March 2013 and October 2015. Fig. 4 provides a chronological view of the request, receipt and central review of patient samples over the 4 years taken for sample collection and review.

**Table 1:** Tissue received at central laboratory and reasons for inability to do Gleason rescore.

*Tissue received at central laboratory*	
No blocks, slides only	40
1 block	310
2 blocks	786
3 blocks	86
4 blocks	144
5–10 blocks	564
11–15 blocks	115
>15 blocks	2
Total	2047

*Reasons for inability to do central Gleason rescore*	
No cancer in material submitted	27
Cutting out, inadequate tissue	77
Discrepant pathology number	4
Not prostate tissue	4
Slides only, no blocks, slides uninterpretable	39
Total	151

### Central pathology review

Core biopsies from 1854 patients and TURP specimens from a further 41 patients underwent central pathology review by a specialist uropathologist. Table 1 outlines reasons why in some cases (n = 151), it was not possible to provide a centrally assigned grade group from core biopsies. Table 2 shows the grade groups assigned for core biopsy specimens from both the recruiting centre pathologist and the ICR-based study-specific uropathologist.

**Table 2:** Comparison of grade group assigned by recruiting centre and after central pathological review.*

Recruiting centre grade group	Grade group after central pathological review						
	1	2	3	4	5	Unknown	Total
**1**	**248**	334	37	5	1	89	714
**2**	126	**520**	138	23	9	68	884
**3**	33	160	**106**	23	34	34	390
**4**	5	16	17	**12**	7	1	58
**5**	0	0	0	0	**0**	0	0
**Unknown**	1	0	0	0	0	0	1
**Total**	413	1030	298	63	51	192	2047
**Comparison of Gleason grading and Grade groups**							
Grade group 1	GS ≤6						
Grade group 2	GS 3 + 4 = 7						
Grade group 3	GS 4 + 3 = 7 (if% grade 3 ≥5%)						
Grade group 4	GS 4 + 4 = 8						
	GS 4 + 3 = 7 (if% grade 3 <5%)						
	GS 3 + 5 = 8						
	GS 5 + 3 = 8						
Grade group 5	GS 4 + 5 = 9						
	GS 5 + 4 = 9						
	GS 5 + 5 = 10						

GS: Gleason score.

* Note core biopsies only, 41 patients providing TURP specimens are not included.

886/1854 (47.8%) patients had the same grade group assigned by the recruiting centre and the study-specific uropathologist. For 357/1854 (19.3%), a higher grade group was assigned at the recruiting centre than following central review. For 611/1854 (33.0%), a lower grade group was assigned at the recruiting centre than following central review. For 163/1854 (8.8%) of patients the difference in grade group was two or more points. Patients with an overall Gleason score of 9 or 10 (grade group 5) were not eligible to enter the CHHiP trial however 51 patients were assigned grade group 5 following central pathological review. Of the 2047 Trans-CHHiP patients, 371/625 (59.4%) of NCCN low risk patients would have been reclassified as intermediate risk, 6/625 (1.0%) of NCCN low risk patients would have been reclassified as high risk and 89/1172 (7.6%) of NCCN intermediate risk patients would have been reclassified as high risk

Importantly, the pathologist at the treating centre, and the central specialist uropathologist did not always review identical tumour sections for each case and therefore may have observed biologically different tumours. Additionally, there was often several years between the two different pathological reviews and grade shift in an upward direction over the last decade is well-recognised. Overall the number of patients who had a change in grade group illustrates the importance of central pathological review in translational studies. Trans-CHHiP also provides an opportunity to further validate the grade groups by assessing survival outcomes [4], this work is ongoing.

The Gleason score and assigned grade groups have a subjective component [7], which is minimised by central pathology review. The initial use of samples has been for an immunohistochemistry study. This study has a case:control design where grade group is one of the matching criteria used to identify control patients, therefore minimising subjectivity is important. In Trans-CHHiP, time-consuming detailed histopathological review was dependent on recruitment of a recently retired highly experienced specialist uropathologist. In our experience, a limiting step in biomarker research is finding an experienced pathologist with available time.

### Lessons learned

We are extremely grateful to the patients and centres within CHHiP for donation of 2047 samples. Immunohistochemical and genomic studies are currently underway which we hope will yield clinically useful results. The high number of samples obtained substantially increases the statistical robustness of these studies. Two aspects of the sample collection methodology were crucial for the successful receipt of over two thousand samples, in addition to the generosity of patients and trial centres. Firstly, a highly motivated CTU-based team organising the MTA, sending sample requests and repeated reminders. Secondly, regular meetings of the central Trans-CHHiP group for feedback, resolution of problems and setting of milestones.

Several factors prevented request of samples. This included lack of consent for Trans-CHHiP, often from patients in Part I of the trial where the main consent form did not include a clause relating to tissue collection. We therefore recommend consent for translational work being obtained on the same form as the main trial consent form. The decision to exclude international centres from Trans-CHHiP, for legal and contract-related reasons, also reduced the number of samples requested. Thirdly a lack of an integral MTA within the clinical trial site agreement significantly delayed sample receipt because a number of centres requested renegotiation of the original site agreement. In future studies we suggest the MTA is included as standard within the original site agreement (even if sample collection will not happen for some time).

Further factors prevented receipt of samples once they had been requested. A small number of centres demanded more financial remuneration than we had budgeted to pay. Of pathology departments providing invoices, 89 of 95 (93.7%) were paid the anticipated £15 per sample however 6 departments were paid a higher sum, which for one department (that provided 42 samples) was more than £50 per sample. 12 departments did not provide any invoices despite chasing, in some cases this was because of the lack of an appropriate pathology department account in which to deposit funding. This suggests a need for improved organisation of financial remuneration for pathology departments, which would also encourage participation in translational research. Provision of funds to support central pathology review is also recommended.

A lack of pathology link person at some centres restricted how effectively we were able to chase up sample requests. Samples may be stored in pathology departments away from the location of the consenting radiotherapy centre; for current translational studies at the ICR-CTSU we are recording where samples are stored alongside the histopathology number for each patient at trial recruitment. A record of when samples are due to be archived elsewhere could also minimise loss of samples as retrieval following archive was usually not possible.

CHHiP was designed prior to “the genomic revolution” during which next-generation genomic technology has become more widely available and affordable. In view of this no germline blood samples were collected. This has not been a substantial limitation but has provided an added complication to planning of DNA-based next-generation sequencing. However we recognise that technological progress cannot be easily anticipated at the start of a randomised study that recruits patients over several years.

## Conclusions

The collection of over two thousand patient samples from 67 centres participating in CHHiP has enabled well-powered histological and genomic translational studies to commence. This article has highlighted aspects of our sample collection methodology that was effective, but also identified areas needing refinement. The recently launched NCRI CM-Path initiative in the United Kingdom will help address academic pathological challenges encountered in conducting translational studies using clinical trial samples [9]. The increase in translational studies as a whole and expansion of molecular sub-typing of tumours both demand improved national and international collaboration to achieve statistically meaningful sample sizes. A scientific and systematic approach to sample collection methodology has become increasingly important.

## Declarations

Ethics approval and consent to participate:

London MREC Committee reference number: 04/MRE02/10.

## Consent for publication

Not applicable.

## Availability of data and material

All data generated or analysed during this study are included in this published article [and its supplementary information files].

## Funding and role of the funding source

Sample collection was funded by a Cancer Research UK Biomarkers and Imaging Discovery and Development Committee (BIDD) grant obtained in 2011. Further funding to support activities at the central receiving laboratory was also provided by Prostate Cancer UK and the Movember Foundation. The CHHiP trial was funded by Cancer Research UK (CRUK/06/016). The investigators acknowledge NHS funding to the NIHR Biomedical Research Centre at The Royal Marsden and The Institute of Cancer Research, London.

The funding bodies for sample collection had no role in the study design; in the collection, analysis and interpretation of data; in the writing of the report; and in the decision to submit the article for publication.

## Authors’ contributions

All authors read and approved the final manuscript.
