# Supplementary material for: Methodology for tissue sample collection within a translational sub-study of the CHHiP trial (CRUK/06/016), a large randomised phase III trial in localised prostate cancer
Source: Clin Transl Radiat Oncol. 2018 Feb 16;10:1–6. doi: 10.1016/j.ctro.2018.02.002 (PMC6008632; doi:10.1016/j.ctro.2018.02.002)
Supplement: Supplementary data 1 [file mmc1.pdf]

Hospitals contributing tissue samples to Trans-CHHiP (number of patients)

Addenbrooke's Hospital (55)
Aintree University Hospital (1)
Alexandra Hospital, Redditch (11)
Barnsley Hospital NHS Foundation Trust (9)
Basingstoke and North Hampshire Hospital (21)
Bedford Hospital NHS Trust (56)
Belfast City Hospital (16)
Birmingham City Hospital (7)
Birmingham Heartlands Hospital (2)
Bradford Royal Infirmary (5)
Bristol Royal Infirmary (1)
Burnley General Hospital (4)
Charing Cross Hospital (5)
Chesterfield Royal Hospital (26)
Countess of Chester Hospital (33)
Croydon University Hospital (88)
Darlington Memorial Hospital (1)
Dewsbury and District Hospital (12)
Doncaster Royal Infirmary (31)
Ealing Hospital, London (4)
East Surrey Hospital (15)
Eastbourne District General Hospital (36)
Epsom Hospital (54)
Falkirk Community Hospital (1)
Frimley Park Hospital (9)
Furness General Hospital (1)
George Eliot Hospital, Nuneaton (4)
Gloucestershire Royal Hospital (1)
Good Hope Hospital, Birmingham (3)
Hereford County Hospital (18)
Hillingdon Hospital (2)
Huddersfield Royal Infirmary (8)
Ipswich Hospital (137)
James Paget University Hospital, Great Yarmouth (1)
Kent and Sussex Hospital (1)
Kettering Hospital (18)
Kingston Hospital (70)
Lincoln County Hospital (150)
Maidstone Hospital (3)
Manchester Royal Infirmary (13)
Milton Keynes University Hospital (10)
Musgrove Park Hospital, Taunton (10)
Newham University Hospital (13)

NHS Fife (1)
NHS Forth Valley (8)
NHS Inverclyde (16)
Noble’s Hospital, Isle of Man (11)
Norfolk & Norwich University Hospital (57)
North Staffs University Hospital (21)
Northampton General Hospital (19)
Northwick Park Hospital, London (29)
Princess Alexandra Hospital, Harlow (11)
QE Gateshead (4)
Queen Elizabeth Hospital, Birmingham (33)
Queen Elizabeth Hospital, King's Lynn (55)
Queen Elizabeth II Hospital (1)
Queen Hospital, Romford (9)
Rochdale Infirmary (1)
Royal Albert Edward Infirmary, Wigan (2)
Royal Blackburn Hospital (30)
Royal Bolton Hospital (14)
Royal Bournemouth Hospital (6)
Royal Free Hospital (2)
Royal Lancaster Infirmary (57)
Royal London Hospital (17)
Royal Marsden Hospital, London (5)
Royal Oldham Hospital (9)
Royal Preston Hospital (13)
Royal Surrey County Hospital, Guildford (16)
Royal Sussex County Hospital, Brighton (43)
Royal United Hospital, Bath (8)
Royal Victoria Hospital, Newcastle (9)
Russell’s Hall Hospital (5)
Salisbury District Hospital (2)
Shirley Oaks Hospital (BMI) (3)
Southern General Hospital (2)
Southmead Hospital (15)
Southport and Formby District General Hospital (48)
St. George's Hospital (22)
St. Helier's Hospital (26)
St. James University Hospital, Leeds (22)
St. Marys Hospital, Paddington (6)
St. Richard’s Hospital, Chichester (1)
Stepping Hill Hospital (6)
Stirling Community Hospital (4)
Tameside General Hospital (7)
The Chase Farm Hospital (2)

Torbay Hospital (5)
Tunbridge Wells Hospital (1)
University Hospital Ayr (10)
University Hospital Coventry (40)
University Hospital Crosshouse (Glasgow) (42)
Wansbeck General Hospital, Northumberland (1)
Warrington Hospital (81)
Warwick Hospital (19)
West Middlesex University Hospital (2)
West Suffolk Hospital (73)
Western General Hospital, Edinburgh (2)
Western Infirmary, Glasgow (4)
Weston General Hospital (2)
Whipp's Cross Hospital (24)
Whiston Hospital (66)
Whittington Hospital (5)
Worcester Royal Hospital (10)
Wrexham Maelor Hospital (1)
Wythenshawe Hospital (6)
Yeovil District Hospital (9)

CHHiP Trial Steering Committee Members

Professor Anthony Zietman, (Chair, Professor of Radiation Oncology, Harvard Medical School) Boston, Massachusetts, USA

Professor Søren M Bentzen (Director of the Division of Biostatistics and Bioinformatics, Director of the UMGCC Biostatistics Shared Service, University of Maryland School of Medicine), Baltimore, USA

Dr Heather Payne, (Consultant in Clinical Oncology), UCLH, London

Dr Vivian Cosgrove, (Head of Radiotherapy Physics), St. James's Hospital, Leeds

CHHiP Trial Management Group Members

Angela Baker, Margaret Bidmead, Ananya Choudhury, Clare Cruickshank, David Dearnaley, John Graham, Clare Griffin, Emma Hall, Shama Hassan, Haley James, Vincent Khoo, Helen Mayles, Philip Mayles, Olivia Naismith, Julia Pugh, Paul Ridley, Christopher Scrase, Chris South, John Staffurth, Isabel Syndikus and Jean Tremlett.

SAMPLE LOCATION FORM

CHHiP Pathology Details Form

Please check that histology number is correct; if incorrect please amend, if no histology number on the form then write in space provided.

**Trial Number**	**Histology Sample Number**

Patient's Initials ☐ ☐

Has this patient consented to the use of their tissue samples for research into prostate cancer? **Yes** ☐ **No** ☐

Has this patient consented to the RAPPER study? **Yes** ☐ **No** ☐

Name of Reporting Consultant Pathologist *(please use block capitals)*

Name of Hospital where the blocks are held (please tick as appropriate)

RMH Sutton ☐ **Epsom** ☐ **Kingston** ☐ **Mayday** ☐

*If other please state:

***If other please provide address details:**

Address *(please use block capitals)*

Postcode ☐☐☐☐☐☐☐

Signed ______________________ **Date** ☐☐☐☐☐☐

Please send completed form to: CHHiP Trials Office,
ICR-CTSU, Sir Richard Doll Building, Cotswold Road, Sutton, Surrey SM2 5NG

CHHiP

Sample Transfer Form: *Trans*CHHiP

Conventional or Hypofractionated High Dose Intensity Modulated Radiotherapy for Prostate Cancer (Translational)

Please complete the form and send back as detailed below.

Trial ID	Initials	Date of Birth	Histology Number	Number of Blocks Sent	Number of Slides Sent	Date Sent

Please send the tissue samples and the completed BLUE copy to:
CHHiP Study (Christine Stuttle)
Clinical Academic Radiotherapy
Male Urological Cancer research Centre (MUCrC)
Institute of Cancer Research and Royal Marsden Hospital
15 Cotswold Road,
Sutton,
Surrey
SM2 5NG
Tel: 020 8722 4464 / 4146
e-mail: christine.stuttle@icr.ac.uk

Please keep the lilac copy for your records.

Please send the completed ORANGE copy of this form to:
CHHiP Trial Manager
ICR Clinical Trials & Statistics Unit (ICR-CTSU)
Division of Clinical Studies
The Institute of Cancer Research
Sir Richard Doll Building
Cotswold Road
Sutton, Surrey
SM2 5NG
Tel: 020 8722 4183
Fax: 020 8770 7876
e-mail: chhip-icrctsu@icr.ac.uk

CHHiP Pathologist Re-score **Box No**

Case reference number   Number of H and E slides

Total number of cores   Number of cores with cancer

	Core1	Core 2	Core 3	Core 4	Normal X
Core reference Number					
Gleason primary pattern					
Gleason secondary pattern					
Tumour involvement (Extensive or Focal)					
Gleason score for focus					
Length of Cancer in core					
Slide Marked (Y/N)					
**CASE ASSESSMENT**					
Overall Gleason score for case					
Maximum length of cancer in individual core					
Maximum % cancer in individual core					
Best core(s) for unstained spares					
Comments					

CHHiP Pathologist Re-score **Box No**

Case reference number | Number of H and E slides

Total number of chips | Number of chips with cancer

	Chip1	Chip2	Chip3	Chip 4	Normal X
Core reference Number					
Gleason primary pattern					
Gleason secondary pattern					
Tumour involvement (Extensive or Focal)					
Gleason score for focus					
Maximum size of focus					
Slide Marked (Y/N)					
**CASE ASSESSMENT**					
Overall Gleason score for case					
Percentage of chips containing cancer					
Best block for unstained spares					
Comments					
